# The Smart Drifter Cluster: Monitoring Sea Currents and Marine Litter Transport Using Consumer IoT Technologies †

**DOI:** 10.3390/s23125467

**Published:** 2023-06-09

**Authors:** Silvia Merlino, Vincenzo Calabrò, Carlotta Giannelli, Lorenzo Marini, Marco Pagliai, Lorenzo Sacco, Marco Bianucci

**Affiliations:** 1 Istituto di Scienze Marine del Consiglio Nazionale delle Ricerche (ISMAR-CNR), 19032 Lerici, SP, Italy; silvia.merlino@sp.ismar.cnr.it; 2 MDM TEAM S.r.l., 50121 Firenze, FI, Italy; vincenzo.calabro@mdmteam.eu (V.C.); lorenzo.marini@mdmteam.eu (L.M.); marco.pagliai@mdmteam.eu (M.P.); 3 Department of Mathematics and Informatics “Ulisse Dini”, University of Florence, 50134 Firenze, FI, Italy; carlotta.giannelli@unifi.it (C.G.); lorenzo.sacco@unifi.it (L.S.)

**Keywords:** Smart Drifter Cluster, marine current, marine drifters, wireless communication, marine litter dispersion, citizen science, IoT

## Abstract

The study of marine Lagrangian transport holds significant importance from a scientific perspective as well as for practical applications such as environmental-pollution responses and prevention (e.g., oil spills, dispersion/accumulation of plastic debris, etc.). In this regard, this concept paper introduces the Smart Drifter Cluster: an innovative approach that leverages modern “consumer” IoT technologies and notions. This approach enables the remote acquisition of information on Lagrangian transport and important ocean variables, similar to standard drifters. However, it offers potential benefits such as reduced hardware costs, minimal maintenance expenses, and significantly lower power consumption compared to systems relying on independent drifters with satellite communication. By combining low power consumption with an optimized, compact integrated marine photovoltaic system, the drifters achieve unlimited operational autonomy. With the introduction of these new characteristics, the Smart Drifter Cluster goes beyond its primary function of mesoscale monitoring of marine currents. It becomes readily applicable to numerous civil applications, including recovering individuals and materials at sea, addressing pollutant spills, and tracking the dispersion of marine litter. An additional advantage of this remote monitoring and sensing system is its open-source hardware and software architecture. This fosters a citizen-science approach, enabling citizens to replicate, utilize, and contribute to the improvement of the system. Thus, within certain constraints of procedures and protocols, citizens can actively contribute to the generation of valuable data in this critical field.

## 1. Introduction

Following the indications of the European Commission in the context of the Integrated Maritime Policy inaugurated by the Blue Book (COM2007(575)), in the coming years, it is desirable to arrive at a system of integrated sea observation and forecasting systems for intermediate and end users (COM2009(544)) that provides data, products, and services to facilitate the management of coastal and marine environments, intervene in the presence of risks, implement security in sensitive areas, and provide visibility on the quality of the coastal and marine environment (EMODNET—European Marine Observation and Data Network). Therefore, expanding and optimizing the communication capabilities between oceanographic measurement instruments can bring significant improvements in the monitoring of ocean currents and search-and-rescue activities in the Mediterranean Sea. In the specific case of research on marine currents, passive buoys (drifters: [1,2,3]) are transported by the surface movements of water or currents within the first few meters. The main types of research drifters are the SVP drifter [4], the CODE drifter ([5,6]), and the CARTHE drifter [7]. They are designed for the study of ocean circulation, plankton dispersion, runoff dispersion, and oil spill modeling. They attempt to minimize Stokes drift and direct wind force (windage) while following the near-surface (from about 0.5 m to 10 m-deep) flow. There are also some drifters, such as the ARGOSPHERE type, designed for hydrocarbon spills that follow the currents in the first few centimeters of water. The usual communication method of these drifters is by satellite (IRIDIUM, Globalstar-SPOT, ARGOS), justified by the fact that they are designed to follow real-time large-scale marine currents, often very far from the coastline. Drifter designs that focus on the transport and dispersion of pollutants such as floating marine debris or oil spills typically have a lower drag area ratio and target the transport of material in the upper meter of water, taking into account wind- and wave-induced motions that primarily influence the transport, for example, of “macro”-plastics [8].

Recent developments of software and hardware technologies in the field of Internet of Things (IoT), pattern recognition, and sensing, offer the possibility to explore new ways to study Lagrangian ocean currents. The basic idea is to create an integrated system for the remote acquisition of marine data using a new conception of “drifters” that are much cheaper and logistically simpler than the current ones by exploiting the latest software and hardware consumer technologies.

Very simple types of these drifters are marine litter trackers (MLTs) [9]. In effect, MLTs are objects representing different types of real “marine litter”, such as wooden boards and plastic bottles of different shapes and volumes. The MLTs do not have the capacity to communicate with each other: they just contain optimized electronics/devices for GSM/LoRa (long-range) communication to land. The cost of MLTs is particularly low (some tens of euros) and they are especially suitable for monitoring marine litter discharged from rivers into the sea, which normally follows paths close to the coastline. Among the innovations introduced with MLTs, compared to other similar systems previously tested [10,11,12,13], is the ability to record data so that, when connected to the GSM/LoRa network, data tracked offline can also be sent. Another key feature of these devices is that they are completely energy independent, with virtually unlimited autonomy, thanks to an appropriate onboard photovoltaic system, which is very small in size due to the very low power consumption of MLTs. The readily available technologies used for MLTs allow for the direct involvement of citizens in the acquisition process in this field as well as raising awareness of related issues (pollution dispersion and marine debris). In fact, for the past three years or so, as part of “alternanza scuola lavoro” projects (“school-work internship”, now identified in Italy by the acronym PCTO), numerous schools have been successfully involved in the utilization of MLTs. Taking a cue from this collaborative approach, we would also like to involve the community of so-called “makers” (Non-specialized people, i.e., not necessarily specialized technicians/engineers, who use popular hardware/software, such as Arduino to make a wide variety of tool/gadgets themselves. This community is becoming very large, as evidenced by the growing number, and the success, of dedicated fairs and websites/forums).

From this CNR-ISMAR experience in developing and testing MLTs came the idea of using low-cost IoT technologies for the more ambitious goal of introducing the cluster concept (the Smart Drifter Cluster) to the mesoscale monitoring of marine currents, with further possible applications for the recovery of people/materials lost at sea and the monitoring of off-shore pollutant spills/marine litter.

In fact, the MARTA project (“SVILUPPO DI UNA INNOVATIVA MULTIPIATTAFORMA SMART DRIFTER–UMV–SAPR PER INDAGINI MARINE”–(M.A.R.T.A.), POR CreO FESR Toscana 2014–2020, Call R&S 2020, Activity 1.1.5.a1. Partners: SIGMA INGEGNERIA S.R.L. (coordinator), MDM TEAM S.R.L., DMG) brings together companies and research institutions with the aim of making the Smart Drifter Cluster idea, developed within CNR-ISMAR, a reality. The final purpose is to create an integrated system for remote, “real-time” data acquisition on marine currents and pollutants in a much cheaper and logistically simpler way than the current ones. For massive production (some thousands of pieces), the target production cost of each drifter is about 200€; the target cost is less than twice this amount. These figures are significantly less than those for standard drifters used in oceanography. In addition, the Smart Drifter Cluster is also designed to minimize the cost of remote data communication. This is achieved by minimizing the use of satellite communication thanks to the introduction of the GSM/G4/LoRa network.

If these objectives are successfully realized, similar to the previous MLTs (Mobile Litter Traps) experience, it will create opportunities to engage citizens in remote marine monitoring/sensing and marine-litter tracking.

## 2. Materials and Methods

The Smart Drifter Cluster is mainly characterized by two different wireless communication types and capabilities, designed according to two different needs: internal data/information exchange among the many drifter elements and external communication to a remote server/user. The former type is used to make the cluster character of the system effective, while the latter is usually reserved for a special drifter element and allows the cluster to be connected to an external, remote receiver.

### 2.1. Development of the Project with Parallel Activities

From a technological standpoint, the project can be divided into multiple components that need to be integrated with each other. Consequently, the implementation of the Smart Drifter Cluster was executed through various activities, each with distinct sub-objectives, while considering the shared overarching goal. The list of the different activities is given hereafter:

1. Research and development (R&D) of an electronic circuit incorporating GPS, GSM, WiFi, and LPWAN communication functionalities aimed at enhancing the long-range capabilities and energy efficiency of cutting-edge, cost-effective wireless communication technologies. The circuit is designed to be optimized for the specific use case.
2. IT for intelligent management of cluster elements and drifter data processing; design of the software that implements the communication strategies among the elements of the drifter cluster and also manages the communication modes (intensity, frequency, synchronizations, data type, etc.).
3. Testing of different physical housing for the drifter made with materials with a low environmental impact and with the appropriate mechanical characteristics appropriate for the specific use of the drifter (semi-submerged, with floating buoy drag, etc.).
4. Design of a propulsion system for the few primary elements to provide a light navigation capacity to maximize data collection from the secondary elements and to maximize long-range communication capabilities.
5. IT (with digital twin technology) for the primary elements to forecast long-term motion and future cluster fragmentation and to develop an optimization policy based on the quality and quantity of collected data.
6. Energy efficiency and energy harvesting to make the smart drifters virtually energy autonomous.

### 2.2. Existing Technologies and Materials Used

For hardware development, we consider GPS receivers with low consumption and low cost and a PSoC (programmable system on a chip) Cypress microcontroller with very low consumption and with power-down capabilities. The optimization of wireless communication was undoubtedly a top priority, as its effectiveness directly influences the success of the project. Thus, apart from an optimized GSM system, attention will be focused on the study of low-power wide-area network (LPWAN) technology, a wireless transmission system designed to cover long distances at a modest bit rate and create a network of objects communicating with each other with low power consumption, reaching data rates between 0.3 kb/s up to a maximum of 50 kb/per channel, differing from WiFi communication, which uses high bit rates and non-negligible power consumption. Among the LPWAN available standards, we decided to use LoRa technology, a CSS (chirp spread spectrum) proprietary technology that uses free sub-gigahertz frequencies (169 Mhz, 433 Mhz, 868 Mhz, and 915 Mhz) that can reach considerable distances up to 15 km depending on the power used and territorial morphology. The rather wide diffusion of this standard among the various manufacturers allows it to have a low production cost compared to other less established standards. The current range of coverage between neighboring units within the Smart Drifter Cluster is an ongoing area of research in this project, as it is influenced by varying weather and marine conditions. Another component included is an SD flash-memory mass storage device to record data from the same drifter element and data from other drifters in the connected cluster. Possible additional hardware components consist of digital temperature sensors in the SMD package, barometers, and hydrophones.

For the development of the software that implements the communication and control strategies of the hardware, we opted for a mesh-based architecture with methods of “dynamic election” of the “contact point” and a solid-state mass storage device for measurement history with a “dynamic reset” for transmission management.

**Figure 1:**
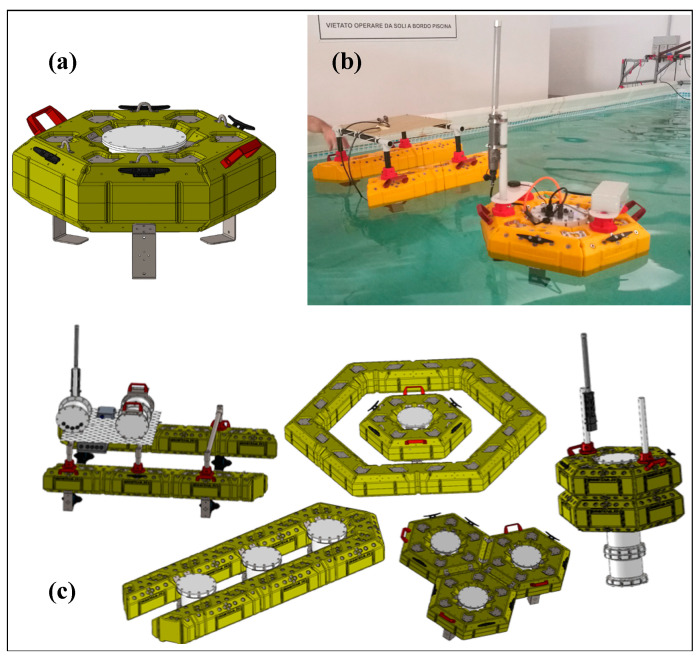
Smart drifter model. (**a**) Standard configuration. (**b**) Photo with two different possible configurations. (**c**) Other possible configurations by assembling the same brick units in different ways (from MDM TEAM s.r.l.—MARTA project).

The floating frame has been designed with the aim of minimizing its environmental impact by utilizing bio-degradable materials. It has also been optimized to meet the requirements of buoyancy and drag imposed by the current, according to the specific needs of its intended use (Figure 1). Thus, part of the project is dedicated to R&D aimed at selecting the most suitable bio-degradable material for the support structure of the drifter, considering its use in the marine environment. The material needs to be strong enough to provide sufficient structural support.

### 2.3. Smart Drifter Cluster: Network Architecture

Within the cluster, the individual drifter element is identified by an electronic code and also a visual element. The elements of the cluster exchange data related first of all to geo-location and possibly other data obtained from environmental devices and sensors installed on the cluster elements. Each element has the dual role of generating and transmitting data related to the same element and bridging the transmission of data from the elements connected to it. Note that, in this way, for the Smart Drifter Cluster to remain connected, it is enough for each element to be able to communicate with its nearest neighbors. A cluster consisting of hundreds of Smart Drifter Clusters can, therefore, remain connected even when dispersion due to sea currents brings the distance between its ends to hundreds of kilometers. When an element of the cluster is intercepted wirelessly by a device capable of bringing information to the user, it is able to provide information about all the elements of the cluster itself.

However, this ideal organization of the integrated network has to contend with the limited bit rate of LPWAN communication technology. This constraint makes it necessary to minimize the amount of data transmitted. It is easy to see that if each element of the Smart Drifter Cluster has to bridge the data transmission of all the other drifter elements in the cluster, the amount of data that each has to transmit increases exponentially with both the number of cluster elements and time. For this reason, it is necessary for each drifter element to have the capacity to partially process its own data in order to “clean” and compact it, thus drastically reducing its size. To do this, it is necessary that the transmission does not take place in real time, with respect to the acquisition of the data, but with a small delay, which is appropriate for transmission in compressed packets.

Another delicate step in network planning is the integration of all outputs from each subsystem (in this case, the remote primary elements) with known or reference data, e.g., obtained from other information sources (satellites, the Internet, etc.) in order to establish a coherent database. This is not a task that can be performed by the drifter elements in the cluster, but will instead be assigned to a centralized processing system located in a ground facility. This is in order to contribute directly to the Pan-European networks, “SeaDataNet, EMODnet, and Copernicus CMEMS”, which provide online integrated marine databases of standardized quality.

### 2.4. Concerning the Probes

First and foremost, it should be emphasized that the primary objective behind the conception and design of the Smart Drifter Cluster is to gather data on the dynamics of surface currents. This is achieved by delivering GPS data that can be transmitted remotely, enabling the tracking of sea navigation routes. The inclusion of other marine monitoring instruments (such as oxygen, salinity, temperature, etc.) on board is considered optional and secondary to the core focus of the project.

However, the project envisages the possibility of installing these instruments, but the constraints of cost (which underlies the idea of the Smart Drifter Cluster) and the small size of the floating elements clearly affect the choice. A part of the MARTA project was, therefore, devoted to comparing, even in the operational phase, different sensors on the market to select those that best meet the cited constraints. Apart from these basic requirements, this selection process also considered the possibility of configuring the hardware and software in an “open” way, i.e., flexible to different needs, as well as ensuring the data quality met the standards for use in an international scientific context.

Based on a preliminary evaluation process, we selected a company that, in our assessment, manufactures and delivers products that meet our requirements [14]. In particular, we chose the sensors shown in Table 1, Table 2, Table 3, Table 4 and Table 5, along with their respective characteristics provided by the manufacturer (Atlas Scientific, Long Island City, NY, USA). We are still in the process of testing the characteristics and calibration of these sensors. Thus, although these results are intriguing, they are preliminary and require further investigation.

## 3. Results

Following what is described in Section 2, a few prototypes of Smart Drifters were developed. The MARTA Smart Drifter is part of a group of floating elements that communicate with each other by transmitting and receiving wireless data, so they collectively behave as a cluster. The suffix “smart” indicates the ability of the drifter to make decisions about different communication strategies (frequency of data sending, type of data sent, signal strength, synchronization process with other elements, etc.) according to different occurrences (rough sea, very expanded cluster, etc.). The MARTA drifter cluster consists of two types of floating units, one simpler and cheaper (the secondary) and the other more sophisticated and expensive (the primary). Almost all the elements in the cluster belong to the secondary type. These are passive in the sense that they have no autonomous navigation capabilities. They are equipped with LPWAN transmission technology, GPS, sensors, SD flash memory, some data elaboration capability, and enough solar panels and storage batteries to ensure unlimited continuous operation time. The few primaries are similar to the secondaries, but they are “active” in the sense that they are equipped with a pair of underwater thrusters and have a more advanced data-processing capacity and long-range communication capability (GSM or satellite for some applications). During regular operations, all the MARTA drifters are moving along with the surface sea current.

### 3.1. Software Architecture

**Figure 2:**
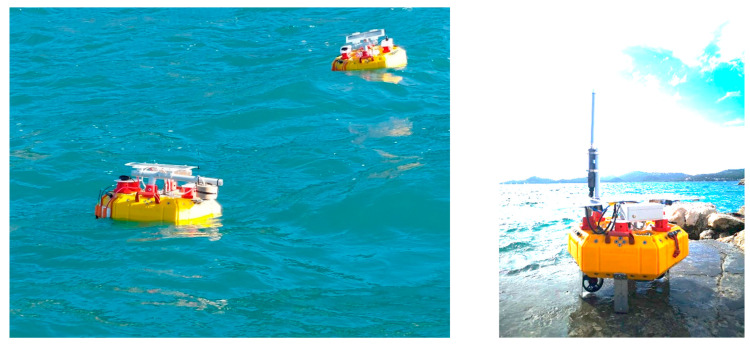
MARTA Smart Drifters prototypes: secondaries on the left and primaries on the right. Note the trusters below the body of the primary type. The final design will have an appropriate shape to minimize direct wind drag.

**Figure 3:**
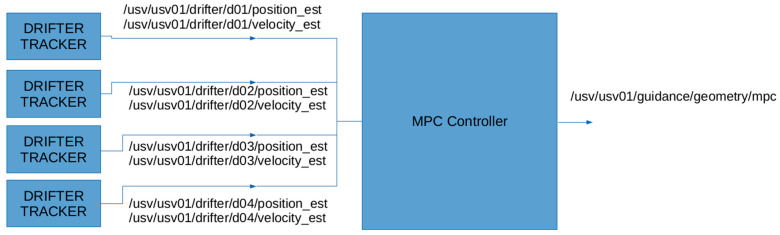
The blue rectangles are the tracker modules of the primary drifter that filter and estimate position and velocity. These values are passed to the blue square that represents the Model Predictive Controller (from MDM TEAM s.r.l.—MARTA project).

All the drifters, both passive and active, share the same mechanical geometry and mass distribution (see Figure 2), despite having different features. This constraint enforces both types to experience the same passive drift. As local turbulence affects drifters to move apart, this could result in dispersion and fragmentation of the initial group of drifters into smaller sub-groups. These may be far enough apart that they cannot communicate with each other via LPWAN. In this case, the initial cluster is no longer fully connected, i.e., it has separated into several smaller, independent sub-clusters. In this situation, the primaries, equipped with active thrusters, will play the role of maintaining external communication coverage with the fragmented clusters. In particular, during regular operation, every drifter will send sensor data measurements and its own geodetic position via radio communication. With this GPS data, the drifter primary will be able to assess the current position of all connected drifters, and thus estimate their actual kinematics. This process is called “drifter tracking” and it is fundamental for the next step named “model predictive control” (Figure 3).

More in detail, during the “drifter tracking”, information is used by each primary drifter to track all drifter positions and velocities on the basis of a kinematic model: assuming a constant irrotational current $v_{c}$, the position p of each passive drifter can be simply modeled on the basis of the kinematic equation

$$\dot{p}=v_{c}$$

Calling $\hat{x}={\left(\hat{p,}\hat{v}\right)}^{T}$ the system of position and velocity estimations, we can predict its discrete evolution considering

$${\dot{\hat{x}}}_{k}=\{\begin{array}{c} {\dot{\hat{p}}}_{k}={\hat{v}}_{k-1}\\ {\dot{\hat{v}}}_{k}=w_{k-1} \end{array}$$

with w∈N(0,Q) normally distributed white noise. The system is updated each time a measured position $\;p_{k}$ is received, through a Luemberger-like filtering

$${\hat{x}}_{k}\leftarrow {\hat{x}}_{k}+K\left(p_{k}-{\hat{p}}_{k}\right)$$

with a *K* matrix of positive parameters to be tuned. The estimated positions and drifts of the secondary units are, therefore, passed to a Model Predictive Controller (MPC) responsible for forecasting long-term motion and possible fragmentation of the cluster. The aim is to use the prediction information to allow primary drifters to autonomously follow the passive vehicles. In the specific, let us consider a scenario with one primary drifter tracking positions ${\hat{p}}_{i}$ and velocities ${\hat{v}}_{i}$ of *N* secondary drifters (Figure 3).

**Figure 4:**
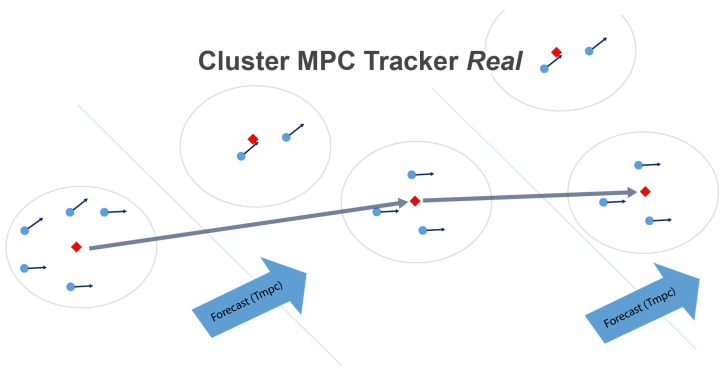
The red rhombus indicates the active drifter (primary) relative to the corresponding connected sub-cluster (the blue circles inside the large circle that includes the primary). Based on the previous position and drift data, the primary elaborates a strategy for the possible activation of the thrusters in order to follow the “assigned” sub-cluster. The estimated positions and drifts of the secondary units are used to forecast long-term motion and future cluster fragmentation. With such a forecasted scenario, the motorized drifters will schedule and communicate to each other its target sub-cluster to follow given a certain optimization policy. (from MDM TEAM s.r.l.—MARTA project).

Once a time instant t and a time interval ΔT have been fixed, the controller predicts the kinematic motion of each drifter as

$${\left(\begin{array}{c} {\hat{p}}_{i}\\ {\hat{v}}_{i} \end{array}\right)}_{k+1}={\left(\begin{array}{c} {\hat{p}}_{i}\\ {\hat{v}}_{i} \end{array}\right)}_{k}+T{\left(\begin{array}{c} {\hat{v}}_{i}\\ 0 \end{array}\right)}_{k},\forall i=1,\ldots ,N,\forall k=1,\ldots ,M$$

with M number of prediction steps. k=1 refers to the estimations received from the tracking modules. $T_{p}=M\Delta T$ is called the prediction horizon. It is now possible to define the set

$$P_{k}={\left\{{\hat{p}}_{1},\ldots ,{\hat{p}}_{N}\right\}}_{k},\forall k=1,\ldots ,M$$

of the predicted positions of all secondary drifters at each time instant t+ΔTk. A hierarchical clustering analysis (HCA) is then applied to each set $P_{k}$ in order to predict the evolution of clusters:

$$\left\{P_{k}\to HCA\to C_{k}^{l},\forall l=1,..,L_{k}\right\},\forall k=1,\ldots ,M$$

where $C_{k}^{l}$ indicates the $L_{k}$ sub-cluster at step *k*. For each subcluster at each time step *k*, the centroid $c_{k}^{l}$ is computed, together with the mean values of the sensor measurements of the passive drifters. We remind that $L_{k}$ could change at some prediction step *k* due to fragmentation/fusion of clusters. Indeed, local turbulence leads each drifter tracker to estimate slightly different drift values for each secondary drifter. On the basis of these prediction scenarios, the motorized drifter schedules its target sub-cluster to follow at each time step given a certain optimization policy:

(1) $$q_{k}=argmin/\underset{l=1,..,L_{k}}{argmax}F\left(C_{k}^{l}\right)$$

where F(  ) is the policy (for example, the sub-cluster with the max number of elements) and $q_{k}$ is the optimal index at step *k*. Once the target sub-clusters are chosen, the set of optimal centroids

$$G=\left\{c_{1}^{q_{1}},\ldots ,c_{M}^{q_{M}}\right\}$$

is used as the interpolation information for the automatic computation of a geometric trajectory to be followed, with a speed given by the average sea current speed of the selected clusters (Figure 4). All of the described prediction procedures are repeated after a time $T_{c}$ called control time, which has to be smaller than the prediction horizon $T_{p}$. We choose $T_{c}=\Delta T$.

In the case of a multi-primary drifter scenario, each primary drifter communicates to the others the first selected optimal sub-cluster $C_{1}^{q_{1}}$ in order to force them to select a different cluster to be followed in the form of constraints inside the optimization problem of (1). This approach will result in a systematic distribution of smart drifters over a finite number of sub-clusters, ensuring long-term data collection, aggregation, and cumulative remote (e.g., satellite) data transfer over time.

The trajectory is computed by the motorized drifter interpolating the forecasted centroids of the chosen clusters, using a specific class of polynomial curves called Pythagorean-Hodograph (PH) [15]. In particular, a polynomial plane curve

$$r\left(u\right)=\left(\begin{array}{c} x\left(u\right)\\ y\left(u\right) \end{array}\right)$$

is a PH curve if and only if

$$x^{\prime }{\left(u\right)}^{2}+y^{\prime }{\left(u\right)}^{2}=\sigma {\left(u\right)}^{2}$$

This means that the norm of the derivative ‖$r^{\prime }\left(u\right)$‖ of the curve with respect to the parameter u is polynomial. This definition leads to interesting computational properties, first of all, the possibility of computing explicitly the length of the curve, being the arc length function

$$s\left(u\right)=\int_{0}^{u}\|r^{\prime }\left(\tau \right)\|d\tau$$

which is also polynomial. As a consequence, it is possible to explicitly compute the time necessary to move along a PH trajectory, given the cruising speed.

**Figure 5:**
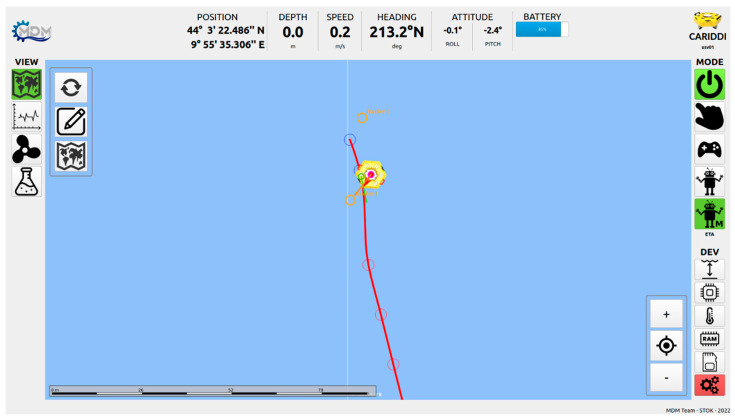
The primary drifter is following the central position of two secondary drifters (orange circles), following a trajectory (red line) computed interpolating predictions of the centroids (circles on the trajectory).

The desired smooth path is constructed by $C^{1}$ Hermite interpolation with PH quintics, see [15,16] and references therein. The properties of smoothness of such a trajectory allow gentle transitions when the configuration of the followed cluster changes, with consequent low-battery consumption. In Figure 5, it is possible to observe an example of computed PH trajectory on the vehicle-control interface, during a real sea trial.

With such a forecasted scenario, the motorized drifters will schedule and communicate to each other its target sub-cluster to follow given a certain optimization policy.

The elements of the MARTA Smart Drifters cluster have been tested in lake and sea trials. In particular, we have validated the quality of the software architecture, with particular attention to the prescription of autonomous trajectories through the prediction module.

### 3.2. Tests

The first batch of tests took place in small lakes due to their moderate environmental conditions. First, we checked and improved the performance of the primary drifter by fine-tuning the system parameters. In particular, it was necessary to do the following:

- verify the water tightness and buoyancy of the vehicles, together with the correct functioning of the two thrusters.
- fine-tune the parameters for the motion estimator, the motion controller, and the guidance law modules responsible for the autonomous following of generic trajectories.
- prescribe a batch of trajectories and verify the ability of the vehicle to follow them. In particular, the path-following error, defined as the error between the geodetic position of the vehicle and the desired geodetic position on the path, had to decrease over time.

**Figure 6:**
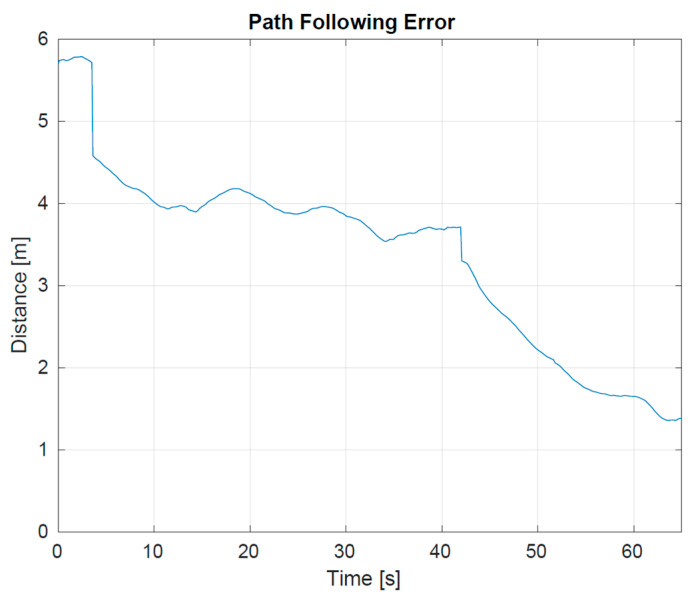
Path following error–norm of error between the geodetic position of the vehicle and the desired geodetic position on the path.

After succeeding in this first phase of testing (see Figure 6), a second set of lake trials was performed with the MARTA Smart Drifters Cluster. In this second scenario, we checked the communication protocol between the vehicles and the capability of primary drifters to track the position and velocity of each secondary drifter, and input information to the MPC module. Moreover, we verified the quality of the measurements collected with the sensors mounted on the drifters and finally the satellite communication.

**Figure 7:**
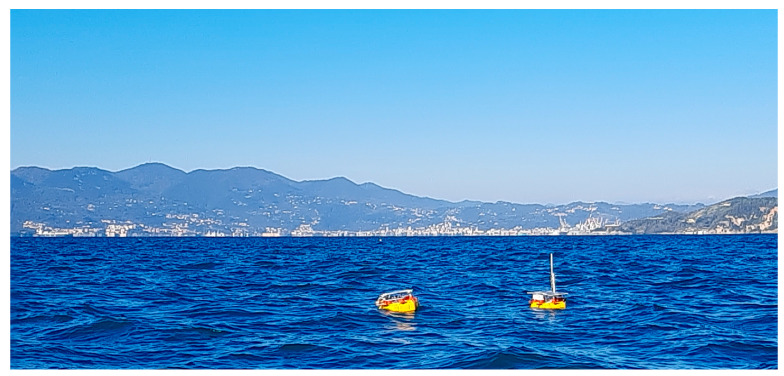
Captured picture of a motorized drifter (on the right) following a single passive drifter (on the left) during the Tellaro sea trial.

**Figure 8:**
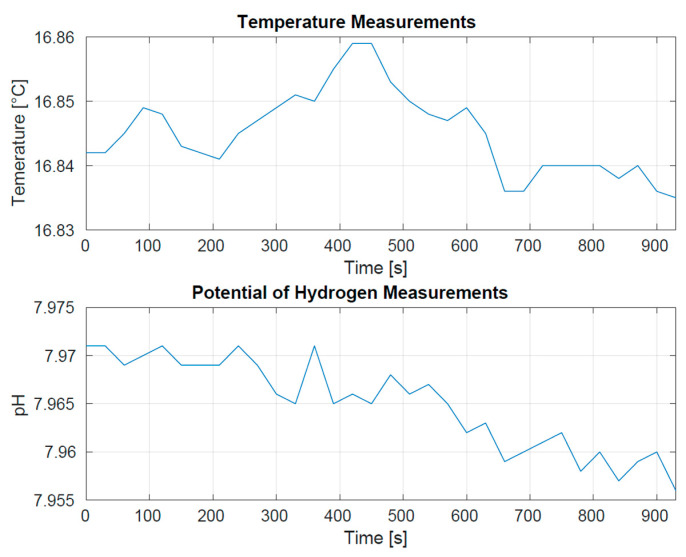
T and pH sensor measurements collected and communicated by a single secondary drifter during the Tellaro sea trial.

**Figure 9:**
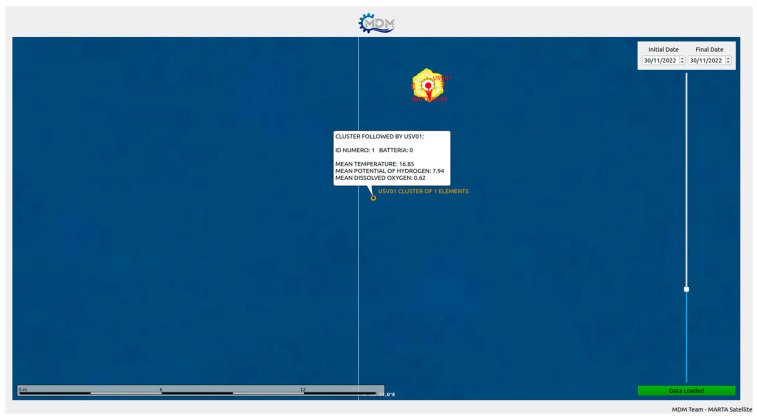
Example of transmitted data, represented on the dedicated GUI. A single primary drifter is following a single secondary drifter (ID 1) with temperature, pH and DO sensors mounted on it.

A final test was held in the sea location of Tellaro, (La Spezia-IT) (Figure 7). On this occasion, we verified that a primary drifter was able to autonomously follow a very small cluster (one and two elements in two different trials) of passive drifters for an extended period. We also verified that each drifter was able to communicate its sensors’ measurements (Figure 8), thus we collected satellite data communications, representing them in real time on a dedicated graphic user interface (Figure 9).

**Figure 10:**
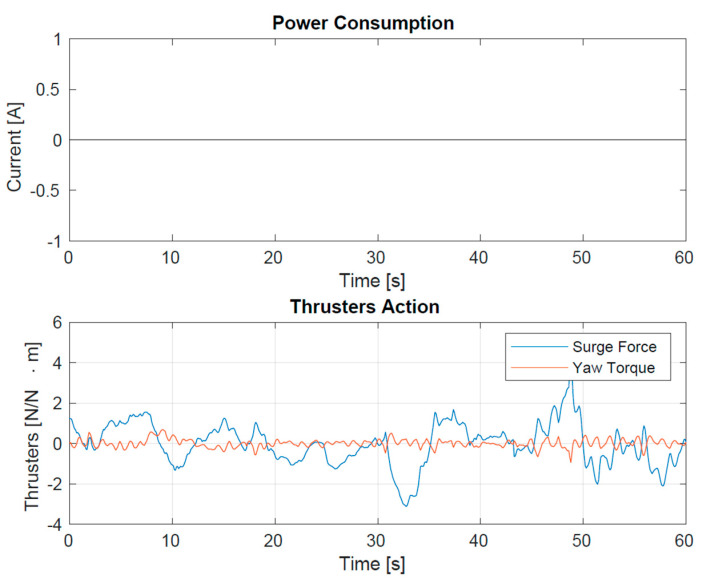
Primary Drifter power consumption during Tellaro (La Spezia-IT) sea trial. The upper plot shows the balance between the dissipated (by the thrusters) and adsorbed (from the PV panels) current. The constant zero value means that it is lower than the threshold of ± 0.2 A. The lower plot shows the corresponding Force and Torque values for the thrusters.

Power consumption and energy harvesting are crucial points in the project. Two lightweight photovoltaic panels, laminated with plastic material (19 cm × 14.5 cm in size, with peak power of 4.5 Wp and MPP voltage of 2.65 V) and made with proven technology for installation in an intensive marine environment, were used. The storage consists of four Li-ion 26650 5000 mAh cells. With this configuration, the drifters turned out to have virtually unlimited autonomy, even in winter and in cloudy conditions. Figure 10 displays an example of the energy balance on board the primary element during one of the sea tests.

In the exceptional case of severe meteorological events extending over time (more than a week), each drifter element is programmed to put itself into a stand-by state as soon as it reaches a power shortage condition. When the energy level of the batteries allows it again, the recorded data is then transmitted on a delayed basis.

## 4. Discussion and Conclusions

An integrated multifunctional sea observation and forecasting system is needed to facilitate the management of the coastal and marine environment, and it is desirable that this system be accessible to a broad class of stakeholders (scientists, policymakers, environmental practitioners, coast guard, marine emergency response services, etc.). In general, one path in this direction is the optimization of communication capabilities between oceanographic measuring instruments. In particular, current developments in sensor, AI, and wireless IoT technologies make it possible to produce low-cost Smart Drifters (linear dimensions on the order of a few tens of centimeters) that, used collectively in large numbers, allow the concept and characteristics of “clusters” to be introduced into marine monitoring. This is the main goal of the MARTA project. In fact, the MARTA Smart Drifter Cluster is a new concept in marine monitoring systems, using drifters that are logistically simpler than current systems based on individual drifters with satellite communication, with lower hardware costs, near-zero maintenance costs, energy harvesting, and reduced power consumption. The suffix “smart” indicates the ability of the cluster’s elements to make decisions about different communication strategies (frequency of data sending, type of data sent, signal strength, synchronization process with other elements, etc.) according to different occurrences (rough sea, very expanded cluster, etc.). The MARTA Smart Drifter Cluster is optimal for sub-mesoscale and mesoscale monitoring of marine currents and for further applications for which other marine monitoring instruments (oxygen, salinity, temperature, etc.) are to be included on board. The entire system, including low-level control of thruster navigation algorithms, radio and satellite communication systems, drifter trackers, and motion predictive control, has been successfully implemented using the popular middleware Robot Operating System 2 (ROS2, https://docs.ros.org/en/foxy/index.html (accessed on 25 May 2023)).

Another goal of the MARTA project concerns the optimization of the mechanical design, constrained by an appropriate and well-studied choice of materials for the support/floating structure of this electronic remote sensing system. This is a daunting task because, at the same time, the structure must meet stringent requirements for dynamic stability, for the ability to passively follow different sea currents (surface and/or directly wind-driven, or sub-surface) depending on different requirements, while minimizing environmental impact. Thus, the materials used must be biodegradable in a marine environment. This latter requirement is a considerable challenge, as recent studies on the degradability of the most common so-called biodegradable materials (PLA, PBAT) show that they degrade rapidly in an environment such as industrial compost, but do not behave in the same way when placed in the marine environment, either in the sea or on the beach [17]. To address this, we are testing a particular blend of corn flour thickeners, which can have degradation times (in a marine environment) of no more than six months.

Some tests have been made to investigate the feasibility of using the wireless communication technology adopted for MARTA smart drifters to study the diffusion (in the sea) and the distribution of accumulation points (on the beaches) of marine debris, akin to the MLTs already quoted in the introduction [9]. This topic is considered, according to ISPRA and the Marine Strategy Framework Directive, one of the main indicators to be monitored in order to define the “Environmental Status” of our seas and coastal areas. Early results have shown that marine litter has a strong dependence on wind rather than on surface sea currents, depending on its shape [9]. This also implies a considerable dispersion of anthropogenic objects transported by rivers once they are released into the sea, and this must be taken into account in the design of Smart Drifter Clusters dedicated to this specific study. The MARTA drifter modular shape (Figure 1) meets these requirements, allowing different configurations: from the classic donut shape (similar to the CARTHE drifters [7]) to other shapes (e.g., catamaran type), with which to simulate the transport of different floating objects. Moreover, again with regard to the study of marine litter, the smart drifters can also be equipped with an innovative sensor (also low-cost and developed within the MARTA project by the CNR-ICCOM) that can count and discern the polymeric type of micro-plastics present on the water surface. This sensor, currently a prototype [18], is being tested in various aquatic environments in order to assess its potential and the reliability of the data obtained. The combination of the Smart Drifter Cluster and the microplastic sensor would introduce a genuinely low-cost monitoring solution in the field of marine monitoring. This approach would bring undeniable advantages, especially when considering the labor-intensive nature of current procedures.

However, we believe that the Smart Drifter Cluster cannot replace the current standard drifters, each equipped with satellite communication, used for studying large ocean currents. The scale of interest for these currents, such as the Gulf Stream or other basin-scale gyres, is extensive, and the related meso and submesoscale dynamics are highly turbulent, resulting in a high probability of fragmentation of the initial cluster into disconnected subclusters. This would require the use of many primary elements (which are more expensive compared to secondaries), negating the overall cost-effectiveness of the system. In the field of marine current studies, the most suitable application for the Smart Drifter Cluster lies in monitoring coastal dynamics. In fact, this project originated from previous campaigns aimed at monitoring the dynamics of marine litter discharged into the sea by rivers.

Our current efforts are focused on expanding and optimizing the communication capabilities of Smart Drifter Clusters. Specifically, we are exploring the use of different high-gain antennas and temporarily increasing the transmission power in LoRa communication. These optimization efforts are focused on expanding the linear cluster size to achieve two main objectives: to decrease the chances of subcluster division and to enhance the likelihood of the cluster boundary being in close proximity to the coast. This proximity enables the utilization of GSM/4G communication, eliminating the need for satellite communication.

## Figures and Tables

**Table 1:** Temperature. PT-1000 Class-A high-purity platinum RTD probe connected to the EZO-RTD Circuit with UART interface. Specifications are provided by the manufacturer.

Optimized Sensing range	0–45 °C
Accuracy	+/−(0.15 + (0.002 × T))
Resolution	0.001 °C
Calibration	Single Point
Data protocol	UART and I2C
Operating voltage	3.3–5.5 V
Size (PCB/probe)	13.97 mm × 20.16 mm/6 mm × 81 cm
Weight (PCB)	1.76 g/40 g

**Table 2:** Conductivity, salinity, and TDS (PPM). Platinum RTD probe connected to the EZO-RTD Circuit with UART interface. Specifications are provided by the manufacturer.

Optimized Sensing range	10–100,000 μS/cm
Response Time	90% in 1 s
Accuracy	+/− 2%
Resolution	0.001
Temperature compensation	Yes
Calibration	2 or 3 point
Data protocol	UART and I2C
Operating voltage	3.3–5.5 V
Size (PCB/probe)	13.97 mm × 20.16 mm/12 mm × 145.5 mm
Weight (PCB/probe)	1.77 g/43 g

**Table 3:** Dissolved Oxygen. This is a quite delicate measure. We have selected a galvanic dissolved oxygen probe consisting of a PTFE membrane, an anode bathed in an electrolyte, and a cathode. Oxygen molecules diffuse through the probes membrane at a constant rate yielding a small voltage at the cathode. The probe is connected to the EZO-RTD Circuit with the UART interface. Specifications are provided by the manufacturer.

Optimized Sensing range	0.01–100 mg/L
Response Time	0.3 mg/L/per s
Accuracy	+/−0.05 mg/L
Resolution	0.001
Calibration	2 points, ~every year
Maintenance	~18 months
Data protocol	UART and I2C
Operating voltage	3.3–5.5 V
Size (PCB/probe)	13.97 mm × 20.16 mm/16.5 mm × 124 mm
Weight (PCB/probe)	1.7 g/52 g

**Table 4:** PH. Silver/silver chloride double junction PH probe connected to the EZO-RTD circuit with UART interface. Specifications are provided by the manufacturer.

Optimized Sensing range	0–14
Response Time	95% in 1 s
Accuracy	+/−0.002
Resolution	0.001
Calibration	1–3 points, ~every year
Data protocol	UART and I2C
Operating voltage	3.3–5.5 V
Size (PCB/probe)	13.97 mm × 20.16 mm/12 mm × 150.6 mm
Weight (PCB/probe)	1.76 g/49 g

**Table 5:** ORP. Silver/silver chloride double junction PH probe connected to the EZO-RTD Circuit with UART interface. Specifications are provided by the manufacturer.

Optimized Sensing range	−2000/+2000 mV
Response Time	95% in 1 s
Accuracy	+/−1 mV
Resolution	0.001
Calibration	Single point, ~every year
Data protocol	UART and I2C
Operating voltage	3.3–5.5 V
Size (PCB/probe)	13.97 mm × 20.16 mm/12 mm × 150.6 mm
Weight (PCB/probe)	1.86 g/49 g

## Data Availability

Data sharing is not applicable to this article.
